# Software- and TDM-Guided Dosing of Meropenem Promises High Rates of Target Attainment in Critically Ill Patients

**DOI:** 10.3390/antibiotics12071112

**Published:** 2023-06-27

**Authors:** Ute Chiriac, Daniel Richter, Otto R. Frey, Anka C. Röhr, Sophia Helbig, Stefan Hagel, Uwe Liebchen, Markus A. Weigand, Alexander Brinkmann

**Affiliations:** 1 Department of Pharmacy, Heidelberg University Hospital, Im Neuenheimer Feld 670, 69120 Heidelberg, Germany; 2 Department of Anesthesiology, Heidelberg University Hospital, Im Neuenheimer Feld 420, 69120 Heidelberg, Germany; 3 Department of Clinical Pharmacy, Heidenheim Hospital, Schlosshaustraße 100, 89522 Heidenheim, Germany; 4 Institute for Infectious Diseases and Infection Control, Jena University Hospital—Friedrich Schiller University Jena, 07740 Jena, Germany; 5 Department of Anaesthesiology, University Hospital LMU Munich, Marchioninistrasse 15, 81377 Munich, Germany; 6 Department of Anesthesiology, Heidenheim Hospital, Schlosshaustraße 100, 89522 Heidenheim, Germany

**Keywords:** meropenem, continuous infusion, dose optimization, therapeutic drug monitoring, dose approximation, pharmacokinetics

## Abstract

Various studies have reported insufficient beta-lactam concentrations in critically ill patients. The optimal dosing strategy for beta-lactams in critically ill patients, particularly in septic patients, is an ongoing matter of discussion. This retrospective study aimed to evaluate the success of software-guided empiric meropenem dosing (CADDy, Calculator to Approximate Drug-Dosing in Dialysis) with subsequent routine meropenem measurements and expert clinical pharmacological interpretations. Adequate therapeutic drug exposure was defined as concentrations of 8–16 mg/L, whereas concentrations of 16–24 mg/L were defined as moderately high and concentrations >24 mg/L as potentially harmful. A total of 91 patients received meropenem as a continuous infusion (229 serum concentrations), of whom 60% achieved 8–16 mg/L, 23% achieved 16–24 mg/L, and 10% achieved unnecessarily high and potentially harmful meropenem concentrations >24 mg/L in the first 48 h using the dosing software. No patient showed concentrations <2 mg/L using the dosing software in the first 48 h. With a subsequent TDM-guided dose adjustment, therapeutic drug exposure was significantly (*p* ≤ 0.05) enhanced to 70%. No patient had meropenem concentrations >24 mg/L with TDM-guided dose adjustments. The combined use of dosing software and consecutive TDM promised a high rate of adequate therapeutic drug exposures of meropenem in patients with sepsis and septic shock.

## 1. Introduction

Beta-lactam antibiotics are the mainstay of therapy for most bacterial infections in intensive care unit (ICU) patients. However, there is growing body of evidence that standard beta-lactam doses either do not achieve the pharmacodynamic targets for killing bacteria (“underdosing”) or are excessive (“overdosing”) in large proportions of critically ill patients, potentially causing harm [1,2,3,4]. One of the main reasons is the high interindividual variability of pharmacokinetic (PK) parameters in critically ill, particularly septic, patients [4,5]. Specifically, hydrophilic drugs, such as beta-lactam antibiotics, show profound pharmacokinetic variabilities in critical illnesses due to capillary leakage and pathophysiological conditions such as altered clearance [5]. Beta-lactams are almost completely cleared by the renal route. Therefore, sepsis-associated transient acute kidney injury (AKI), continuous renal replacement therapy (CRRT), and augmented renal clearance (ARC) due to hyperdynamic states present dynamic challenges to achieve optimal drug exposure [6,7]. An understanding of both the PK changes in critical illness and the pharmacodynamics (PD) of the antimicrobial agent prescribed is essential to ensure the desired drug effects. Optimizing dosing strategies based on accepted PK/PD principles and drug-specific properties are discussed to address this problem [6,7]. These aspects have been underscored by actual international guidelines [8,9]. In this context, software guides such as model-informed precision dosing (MIPD), therapeutic drug monitoring (TDM), and infusion time extension are recommended [8,9,10,11]. Unexpectedly, despite a wide target range of 1–10 × the minimum inhibitory concentration (MIC) in a recent multicenter randomized controlled trial investigating a MIPD/TDM strategy (the DOLPHIN trial), the target attainment remained low, ranging from 56 to 71% [2].

All beta-lactam antibiotics have a time-dependent bacterial killing, and efficacy is related to the percentage of time in the dosing interval, during which the free concentration exceeds the MIC of the target pathogen (% *f*T > MIC). While the in vitro minimum *f*T > MIC value required for static activity ranges from 40 to 70% [12], clinical data in ICU patients show reduced chances of clinical cure, bacteriological eradication, and increased rates of antimicrobial resistances when using the conservative PK/PD target of 40–100% *f*T > MIC [4,13]. Current experimental [14] and clinical [2,6,15] trials use a target of 100% *f*T > 4–8(10) × MIC probably because the concentration in the effect compartment is mostly lower than the blood concentration, and efficacy might be better at higher antibiotic concentrations for preventing the development of resistance [2,14,15]. Conversely, safety issues could emerge if more aggressive PK/PD targets were aimed at [16,17,18,19]. Adverse effects such as neurotoxic and nephrotoxic effects, possibly related to excessively high serum concentrations, have been reported for piperacillin, meropenem, and ceftazidime [20], but are probably underestimated. Furthermore, recent data identified a significantly higher mortality in critically ill patients with piperacillin concentrations of >4–8 × MIC [18,21,22,23]. These data raise the question of whether very high concentrations of meropenem are also associated with higher mortality and how to avoid excessive concentrations of antibiotics.

In 2013, a routine TDM program for meropenem was initiated as part of a coordinated antimicrobial stewardship (AMS) program in the ICU of Heidenheim, an academic teaching hospital. The individual first dose was calculated using the CADDy-tool (calculator to approximate drug dose during dialysis) [7]. The retrospective analysis of this program aimed to evaluate the rate of therapeutic exposure of meropenem concentrations (c_MER_) and clinical outcomes in critically ill patients with individualized doses.

## 2. Results

**Figure 1:**
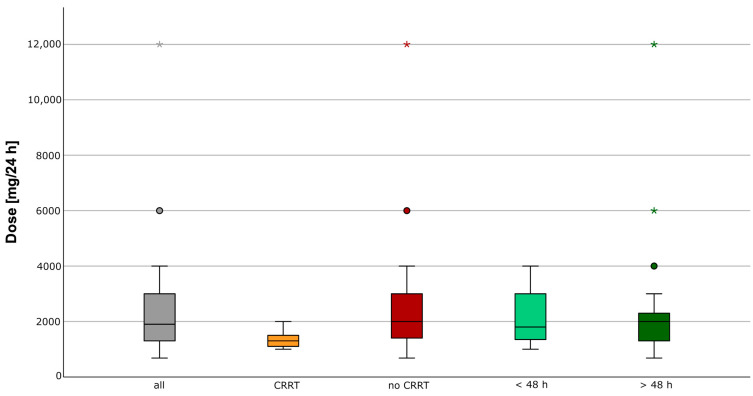
Observed meropenem doses. Distribution of the observed meropenem doses given in mg of all meropenem concentration measurements (*n* = 229), with CRRT (*n* = 21), without RRT (*n* = 208), in the first 48 h (*n* = 91), and after 48 h (*n* = 138). * extreme value, o outlier.

A total of 91 patients were empirically treated with meropenem and included in the study. The study population had a median age of 73 (IQR 18) years with impaired renal function (median CrCL 45 mL/min, IQR 49 mL/min) and CRRT in 12 (13%) patients at admission. Detailed demographic characteristics are summarized in Table 1. Diagnosis and bacterial pathogen distributions are presented in Table 2 and Table S1 in the supplement. The median (IQR) daily dose of meropenem was 1900 (1700) mg (software-guided empiric daily dose: 1800 (1700) mg, TDM-guided daily dose: 2000 (1200) mg), which was less than the standard daily dose of meropenem in these patients, 3000 mg (2000) mg (Figure 1). The dosing range was smaller in patients with CRRT compared to patients without CRRT (Figure 1).

**Figure 2:**
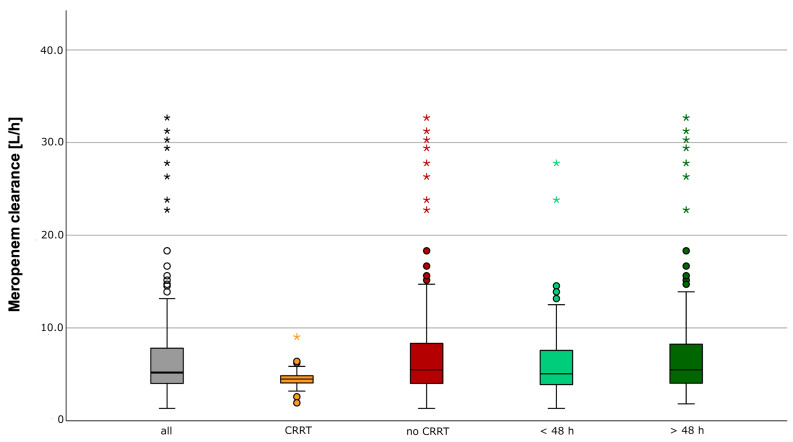
Observed meropenem clearance. Distribution of the observed meropenem clearance given in L/h of all meropenem concentration measurements (*n* = 229), with CRRT (*n* = 21), without RRT (*n* = 208), in the first 48 h (*n* = 91), and after 48 h (*n* = 138). * extreme value, o outlier.

**Figure 3:**
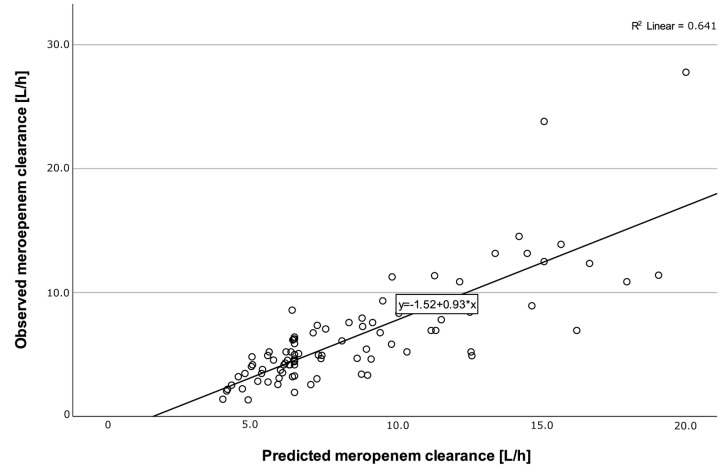
Meropenem clearance predicted by CADDy (CL_CADDY_) versus observed meropenem clearance (CL_MER48_) within the first 48 h (r^2^ = 0.641). The solid black line shows the linear regression line of fit. Correlation was assessed by calculating a Pearson correlation coefficient (r = 0.88).

A total of 229 meropenem concentrations (c_MER_) were measured from the 91 included patients. The median (IQR) c_MER_ was 14.1 (6.0) mg/L (c_MER_ after software-guided empiric daily dosing: 15.6 (7.5) mg/L, c_MER_ after TDM-guided daily dosing: 13.1 (5.5) mg/L). The median observed meropenem clearance (CL_MER_) was 5.2 (3.8) L/h (Figure 2). No ARC was observed (Figure 2) during the first 48 h of treatment. The median meropenem clearance predicted by CADDy (CL_CADDy_) was higher than the observed clearance (CL_MER48_) in the first 48 h (CL_CADDy_ 7.1 (4.2) L/h vs. CL_MER48_ 5.1 (3.8) L/h). The predictive performance of CADDy was assessed by comparing the predicted clearance with the observed clearance (Figure 3).

### 2.1. Therapeutic Exposure

With CADDy-guided dosing in the first 48 h of treatment, therapeutic exposure (8–16 mg/L) was achieved in 60% of patients, moderately high concentrations (16–24 mg/L) in 23% of the patients, and potentially harmful concentrations (>24 mg/L) in 10% of the patients. No patient had meropenem concentrations below the MIC. With subsequent TDM-guided dose adjustments, therapeutic drug exposure was significantly (*p* ≤ 0.05) increased to 70%, whereas no patient had potentially harmful concentrations (>24 mg/L) after TDM-guided dose adjustments (Table 3). In patients with CRRT, we observed a small concentration range with an individualized dosing strategy, of which 9 out of 11 patients (82%) achieved therapeutic exposure with CADDy-guided dosing and 9 out of 10 patients (90%) after TDM-guided dose adjustments. Data describing patients with CRRT are shown in the supplement (Table S2).

Our simulations revealed that standard dosing administered as an intermittent bolus would have achieved therapeutic exposure in 27% of patients with meropenem trough concentrations >24 mg/L in 11% of the patients and <2 mg/L in 20% of the patients. Continuous infusion notably increased therapeutic exposure compared to the bolus application. The effects of the infusion time and the individualized dosing on therapeutic exposure are shown in Table 4. CADDy-guided dosing at the start of the meropenem treatment improved therapeutic drug exposure compared with standard dosing. Details on empiric dosing are described in the supplement (Table S3).

### 2.2. Predictors for Clinical Outcome

**Figure 4:**
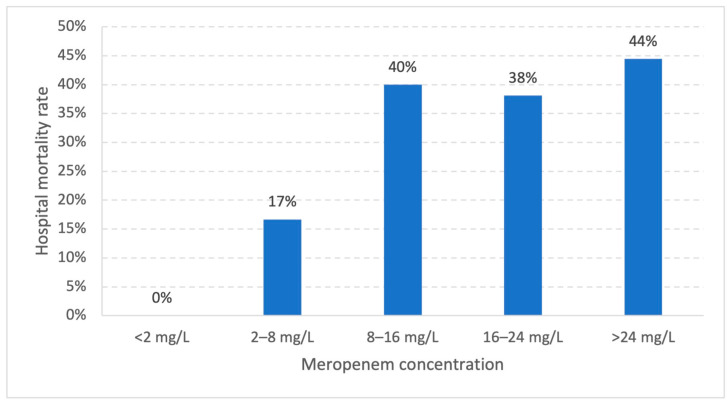
Distribution of hospital mortality rates.

Binary logistic regression revealed that a high SOFA score (OR 1.161, 95% CI 1.057–1.274, *p* = 0.002) and SAPS score (OR 1.055, 95% CI 1.009–1.103, *p* = 0.020) were associated with a significant increase in the odds for hospital mortality. There was no significant association of hospital mortality with c_MER_, age, body mass index (BMI), creatinine clearance (CrCL), or CL_MER_. Hospital mortality in patients with c_MER_ > 24 mg/L (4/9; 44%) (OR 1.200, 95% CI 0.290–4.970, *p* = 0.801) and in patients with 8–16 mg/L (22/55; 40%) was not significantly different (Table 5, Figure 4).

Hospital mortality rates versus meropenem concentrations within 48 h after onset of treatment. Statistical analysis was performed using the chi-square test. Significant levels were considered as *p* ≤ 0.05.

## 3. Discussion

The individualized dosing strategy in this monitor program in ICU patients led to high target attainment rates. In contrast to current MIPD data [2], we achieved our relatively narrow therapeutic target range of 100% *f*T > 4–8 × MIC in 60% of patients within the first 24–48 h, based on software-guided dose adjustments. This was significantly increased to 70% when a subsequent TDM-guided dose adjustment was performed. Our data do not support previous postulations [4,24,25] of insufficiently low serum concentrations associated with sepsis and septic shock within the first 24–48 h, which are often used to justify the administration of excessively high doses of meropenem at the initiation of therapy. At the same time, unnecessarily high meropenem concentrations (c_MER_ > 24 mg/L) occurred in only 10% of patients with empiric dosing and could be completely avoided by dose adjustment with TDM.

Sepsis and septic shock are associated with a high mortality and morbidity rate in critically ill patients [9,10]. Although the target attainment of antibiotics is associated with a positive clinical outcome and with faster resolution of infections in critically ill patients [18,23,26,27], there are a lack of comprehensive outcome data in septic patients treated with software- and TDM-guided antibiotic therapy. One reason might be that sepsis comprises a very heterogeneous group of patients. Other reasons may be structural issues that reduce the likelihood of target attainment in a subset of patients such as restrictive dose adjustments (maximum cut-off for dose increase) or delayed informed consent prior to participation in early dosing interventions. However, traditional antibiotic dosing is known to have repeatedly failed to attain PK/PD targets in previous studies in critically ill patients, which is associated with reduced chances of clinical cure, bacteriological eradication, and an increased risk of antimicrobial resistance [2,4,18,28]. In the DALI trial [4], a prospective multinational point prevalence study of 361 critically ill patients treated with a beta-lactam, only 67% of the patients achieved 100% *f*T > MIC. This was similar to the DOLPHIN trial [2], a recent multicenter randomized controlled trial that included 388 critically ill patients treated with a beta-lactam or a quinolone. With standard doses during the initial course of treatment, only 56% of the patients achieved concentrations within a broad target range of 100% *f*T > 1–10 × MIC and remained low in patients with subsequent MIPD/TDM ranging from 60 to 71% [2]. However, PK/PD targets and the implementation of MIPD in this trial need to be critically reviewed. Given the wide target range, the results suggest a lack of individual dose adjustment. Moreover, it is extremely important to interpret the data considering the mode of antibiotic administration, as most patients received intermittent administration. While intermittent infusion results in unnecessary high peak concentrations followed by low concentrations for a considerable part of the dosing interval [29], prolonged infusion modes are advantageous, as they promise higher beta-lactam concentrations at the end of the dosing interval [16,30,31,32]. If patients in our study had received standard doses as a bolus infusion, most patients would also have shown poor maintenance of effective concentrations. Extending the infusion time to 24 h would have increased the attainment of effective concentrations (100% *f*T > 4–8 × MIC) from 27 to 48%. Applying the individualized dosing strategy based on the PK/PD principles, 66% of the patients achieved adequate therapeutic exposure, and, in addition, a lower proportion had potentially harmful concentrations (standard dosing as CI: 19% vs. individualized dosing as CI: 4%). In contrast, the recent TARGET study realized therapeutic drug exposure of 100% *f*T > 4–8 × MIC only in one third of the patients with TDM-guided dose adjustments of piperacillin administered as a continuous infusion but without software-guided empiric dosing.

In addition to high rates of target attainment, low rates of toxic concentrations are criteria for an appropriate dosing strategy in ICU patients. Higher concentrations, reflecting higher thresholds, might be necessary for preventing resistance development than might be necessary for clinical efficacy [33]. Conversely, it should be recognized that safety issues may arise if more aggressive PK/PD targets are attained [16,17,19]. A recent review emphasized that the majority of clinical pharmacodynamic studies support pre-clinical thresholds and that an exposure between 50 and 100% *f*T > MIC is sufficient for most infections [25]. Furthermore, the MIC distribution in German ICU patients showed some deviation of the local susceptibility pattern from the MIC values reported by EUCAST, allowing the target to be reduced in a similar setting [34]. Harmful effects such as worsening neurological status, potentially related to excessively high serum concentrations, have been reported for piperacillin, meropenem, and cefepime [20,35,36]. Beumier et al. [35] observed neurotoxicity in approximately 40% of critically ill patients with increasing trough concentrations and, therefore, suggested that a c_min_/MIC > 8 for Pseudomonas aeruginosa (i.e., >16 mg/L for meropenem) may expose patients to potential drug toxicity without any apparent benefit in the clinical response to infection. After TDM-guided dose adjustment in this study, no patient had a potentially harmful meropenem concentration above 24 mg/L, whereas 29–44% of the patients in the DOLPHIN trial had a beta-lactam concentration outside the target range (2–20 mg/L) after TDM [2]. A previous German analysis on a TDM-program observed 51% of the patients with meropenem concentrations >40 mg/L, due to insufficient knowledge of dose adjustment [1]. The interprofessional approach to exposure optimization, including expert clinical pharmacological interpretation by trained clinical pharmacists, may have contributed to higher target achievements in our study. Furthermore, the observed meropenem concentrations were obtained in clinical routine through an implemented TDM program and not within a prospective study.

Given the mortality benefits associated with the use of early effective antimicrobial therapy in sepsis [9,10], it is also important that dose individualization occurs before blood samples are drawn for TDM, ideally as part of the first dose. The implementation of empiric dose adjustment, according to renal function employing the CADDy software in our study prior to concentration measurement, might be a useful strategy to avoid either high concentrations at high doses or decreased drug clearance. Unsurprisingly, and in agreement with previous studies, we identified a correlation between renal function and beta-lactam concentrations. We also observed a high variability in renal clearance, including ARC, CRRT, and AKI, as described in critical illness [7,16]. ARC, as well as a reduced clearance in sepsis-associated AKI, should be considered as a risk factor for target failure in antibiotic dosing [7,16]. A number of published studies have postulated a normal or even augmented beta-lactam clearance independent of the renal function in critically ill patients at the onset of infection, resulting in underdosing and recommended to defer renal-dose adjustments within the first 48–72 h of treatment to minimize the risk of underexposure [24,37]. Likewise, in critically ill patients treated with CRRT, antibiotic concentrations varied widely and failed to meet therapeutic targets in a high number of patients [38]. However, our data and another previously published study do not support this point of view [39]. A higher dose requirement during the first 48 h of therapy was not observed. Given the observed variability in critically ill patients, dose adjustment according to renal function using the dosing software might help to avoid potentially harmful effects of very high beta-lactam concentrations, in patients with impaired renal function in particular [16,17,26]. We also observed high rates of target attainment in these patients compared to those reported in the literature [2,3,4] and suggest that dose adjustment according to renal function or CRRT parameters be used from the onset of therapy to avoid very high and subtherapeutic meropenem concentrations.

Several studies found significantly higher mortality rates in critically ill patients with very high piperacillin concentrations, based on forming an outcome group [18,21,22,23]. The authors recommended that high concentrations should be avoided due to the lack of benefits and the potential harmful effects [18,21,22,23]. Forming an outcome group is an innovative approach to find an appropriate target for infection resolution and a better outcome in patients treated with beta-lactam antibiotics, whereas most recommendations are based on animal models or experimental studies or refer to pathophysiological considerations. In our study, we did not observe significantly different hospital mortality rates in patients with very high meropenem concentrations. However, the group with potentially harmful concentrations was quite small (>24 mg/L: 9 patients).

Considering higher rates of target attainment, our study strongly supports the current recommendation for individualized beta-lactam dosing in critically ill patients with sepsis and septic shock. Our results suggest that establishing a dedicated and coordinated multidisciplinary team, renal-dose adjusted empiric dosing, and implementing a real-time TDM-guided individualized antimicrobial exposure optimization method based on expert clinical pharmacological interpretation could be crucial cornerstones for the proper management of sepsis and septic shock in critically ill patients.

There were several limitations to the present study. First, the study was a single-center study with a relatively small sample size (*n* = 91), which may have hindered robust estimates of the extent of PK variability. Due to the retrospective setting, the CrCL was estimated using the Cockcroft–Gault equation, as the CrCL measurement is not performed in routine clinical care. Furthermore, non-renal clearance and organ dysfunction (other than renal) may be relevant but were not considered in the CADDy calculation and might have explained the observed bias in the CADDy-predicted meropenem clearance. Finally, the data set represented a picture of everyday clinical life in intensive care units, and mortality data should be interpreted with caution because of possible confounding factors. An unlimited application to other patients was not possible, and prospective data should be generated. However, retrospective analysis of serum concentrations has the advantage of including all patients, even those with low survival rates, and may provide a more realistic picture as compared to prospective approaches.

## 4. Material and Methods

### 4.1. Study Design and Population

This was a retrospective observational study at a German academic teaching hospital. Ethical approval was waived by the ethics committee of the University of Ulm, Germany (project number 137/19). All critically ill patients admitted to the ICU were screened, and patients ≥18 years of age with sepsis, severe sepsis, or septic shock, according to the definitions of the Surviving Sepsis Campaign (SSC) valid at this time [40], and treated with meropenem administered as continuous infusion were included in this study. Patients were excluded if they were <18 years of age or were treated with meropenem administered by intermittent infusion.

### 4.2. Therapeutic Drug Monitoring (TDM) Program

**Figure 5:**
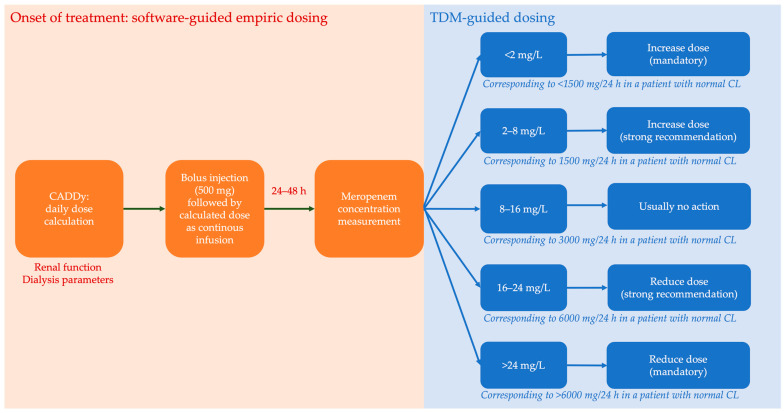
Therapeutic drug monitoring (TDM) program. Individualized dosing procedures including renal-dose-adjusted empiric dosing using CADDy (calculator to approximate drug dose during dialysis) and subsequent concentration measurements. Therapeutic drug exposure was defined as c_MER_ of 8–16 mg/L.

Meropenem was administered to all patients according to our standard operating procedure. This approach consisted of a loading dose (500 mg, 15 min infusion), followed by an immediate continuous infusion with an empirical dose estimated by the CADDy-calculator and subsequently adjusted by TDM within the first 24–48 h (Figure 5). To ensure drug stability, infusions of 20 mg/L in 0.9% sodium chloride were changed after 18 h at the latest [41]. The CADDy program (https://www.thecaddy.de, accessed on 4 September 2023; Dr. Otto Frey, Klinikum Heidenheim) was used to predict meropenem clearance (CL_CADDy_) and meropenem doses in patients. In brief, the CADDy program uses a one-compartment model based on PK population estimates and the Dettli method to calculate meropenem doses, considering CrCL and dialysis settings if applicable [7]. The CrCl was calculated using the Cockcroft–Gault equation [42]. TDM-guided dose adjustments were based on expert clinical pharmacological interpretations by trained clinical pharmacists. CMER was measured using a validated high-performance liquid chromatography (HPLC) assay [43].

### 4.3. Therapeutic Drug Exposure

Therapeutic drug exposure was defined based on the epidemiological cut-off values (ECOFF) of expected pathogens. The ECOFF describes the highest MIC for organisms devoid of phenotypically detectable acquired resistance mechanisms: the upper end of the wild-type distribution (Committee ES (2021) MIC distributions and the setting of epidemiological cut-off (ECOFF) values. https://mic.eucast.org/search/, accessed on 4 September 2023). For adequate therapeutic drug exposure, c_MER_ of 8–16 mg/L, corresponding to four to eight times the ECOFF of *Pseudomonas aeruginosa* (PSA) (http://www.eucast.org/clinical_breakpoints, accessed on 4 September 2023), was used as a rational consensus between effective bacterial killing and potentially harmful concentrations. Meropenem concentrations of 16–24 mg/L were defined as moderately high but acceptable for *Acinetobacter* (ECOFF 4 mg/L), extended spectrum beta-lactamases (ESBL), and deep-seated infections such as infections of the central nervous system. Meropenem concentrations of >24 mg/L were defined as potentially harmful concentrations. To assess the success of software-guided empirical dosing, meropenem concentrations were stratified by the time of observation. In addition, individualized dosing was compared to continuous infusion (24 h) and intermittent bolus administration (0.5 h) of the approved doses according to the German summary of product characteristics (SmPC), as described in Table S4 of the supplement [44]. PK analyses were then performed using a one-compartment model. Concentrations were predicted with Simulx 2023R1 (Lixoft SAS, a Simulations Plus company) using the observed CL_MER_, whereas CL_MER_ was derived using the following equation: ${CL}_{MER}\left[L/h\right]=\frac{dose[mg]}{24h}\cdot {c_{MER}}^{-1}\left[mg/L\right]$, with c_MER_ being the measured meropenem concentration. Details of the model are described in the supplement (Figure S1).

Five different concentration groups (<2, 2–8, 8–16, 16–24, and >24 mg/L) were created to represent the distribution of clinical parameters in relation to c_MER_ within the first 48 h of treatment, and factors likely to contribute to hospital mortality were analyzed for association based on clinical relevance or previously described relationships [18,20,45,46]. These included patient characteristics (age, BMI), disease severity scores (SOFA, SAPS), c_MER_, serum creatinine, CrCL, and CL_MER_.

### 4.4. Statistical Analysis

All calculations and statistical analyses were performed using IBM SPSS Statistics version 26 software (IBM, Armonk, NY, USA). Discrete variables are expressed as counts (percentage) and continuous variables as means ± standard deviation (SD) or median with the interquartile range (IQR). The coefficient of correlation (r) was calculated using a Pearson correlation analysis. Logistic regression analysis was used to evaluate the association of clinical outcomes with patient characteristics. Differences between groups were assessed for statistical significances using the Kruskal–Wallis test for continuous variables and the chi-square test or Fisher`s exact test for categorical variables. Significant levels were considered as *p* ≤ 0.05. We reported median errors, as they were not normally distributed, according to the alternative method of Sheiner and Beal [47].

## 5. Conclusions

Our data strongly supported the use of individualized antibiotic dosing to ensure efficient therapeutic drug exposure in critically ill patients with sepsis and septic shock. The individualized dosing strategy, including continuous infusion, dosing software, and TDM based on expert clinical pharmacological interpretations, led to high rates of target attainment. However, further validation of the dosing software in a clinical trial is required.

## 6. Key Messages

- Meropenem clearance in critically ill patients with septic patients shows high variability.
- Dosing software (CADDy) and TDM allow an approach based on expert clinical pharmacological interpretation, resulting in efficient serum concentrations.
- In contrast to piperacillin, no association between high serum levels and mortality was observed.

## Figures and Tables

**Table 1:** Patient characteristics at admission.

	Median (IQR), no. (%)
Age, years	73 (18)
Weight, kg	80 (20)
Height, cm	170 (10)
BMI, kg/m^2^	27.1 (6.7)
Sex, male	60 (66%)
Creatinine, mg/dL	1.3 (1.6)
CrCL, mL/min	45.3 (53.7)
CRRT	12 (13%)
Mechanical ventilation	54 (59%)
SOFA	8 (8)
SAPS	41 (18)
ICU mortality	28 (31%)
Hospital mortality	35 (39%)
Length of hospital stay, days	26 (28)
Length of ICU stay, days	10 (15)
Antimicrobial treatment, days	7 (4)

BMI: body mass index; CrCL: creatinine clearance; CRRT: continuous renal replacement therapy; ICU: intensive care unit; SAPS: simplified acute physiology score; SOFA: sequential organ failure assessment. Values are given in absolute numbers (N) and relative incidence (%).

**Table 2:** Diagnosis at admission.

Diagnosis	No. (%)
Sepsis/Severe sepsis	55 (60%)
Septic shock	36 (40%)
**Suspected site of infection**	
Pneumonia	34 (37%)
Abdominal infection, peritonitis	12 (13%)
Soft tissue/bone infection	17 (19%)
Urinary tract infection	4 (4%)
Blood stream infection	2 (2%)
Cholecystitis, cholangitis	4 (4%)
Diverse	1 (1%)

Values are given in absolute numbers (N) and relative incidence (%).

**Table 3:** Observed meropenem concentrations stratified by the time of observation. Distribution of meropenem concentrations (c_MER_) in critically ill patients with an individualized dosing strategy within 48 h (=c_MER_ based on software-guided empiric dosing) and after 48 h (=c_MER_ based on TDM-guided dosing).

c_MER_ (mg/L)	<2	2–8	8–16	16–24	>24
Software-guided empiric dosing (*n* = 91)	0 (0.0%)	6 (6.6%)	55 (60.4%)	21 (23.1%)	9 (9.9%)
TDM-guided dosing (*n* = 138)	0 (0.0%)	13 (9.4%)	96 (69.6%)	29 (21.0%)	0 (0.0%)

c_MER_: meropenem concentration; TDM: therapeutic drug monitoring. Values are given in absolute numbers (N) and relative incidence (%).

**Table 4:** Effect of individualized dosing on therapeutic exposure. Distribution of meropenem concentrations (c_MER_) in 91 critically ill patients with a continuous infusion individualized by dosing software and subsequent TDM (=c_MER_ observed based on individualized dosing) compared to continuous infusion without software and subsequent TDM-guided dosing, as well as intermittent bolus administration of standard doses, according to the summary of product characteristics (=c_MER_ predicted based on standard dosing).

c_MER_ (mg/L)	<2	2–8	8–16	16–24	>24
Predicted based on standard dosing (Bolus)	45 (19.7%)	62 (27.0%)	61 (26.6%)	35 (15.3%)	26 (11.4%)
Predicted based on standard dosing (CI)	0 (0.0%)	15 (6.6%)	110 (48.0%)	60 (26.2%)	44 (19.2%)
Observed individualized dosing (CI)	0 (0.0%)	19 (8.3%)	151 (65.9%)	50 (21.8%)	9 (3.9%)

c_MER_: meropenem concentration; CI: continuous infusion. Values are given in absolute numbers (N) and relative incidence (%).

**Table 5:** Cross table depicting the distribution of clinical parameters in different meropenem concentration (c_MER_) groups compared to clinical parameters in patients with therapeutic drug exposure. Statistical analysis was performed using the chi-square and Kruskal–Wallis tests. Significant levels were considered as *p* ≤ 0.05.

c_MER_ (mg/L)	8–16	<2	2–8	16–24	>24
Patients (%)	55 (60.4%)	0 (0.0%)	6 (6.6%)	21 (23.1%)	9 (9.9%)
Hospital mortality (%)	22 (40%)	0 (0)	1 (17%) *	8 (38%)	4 (44%)
Median SOFA score (IQR)	6 (9)	0 (0)	1 (5) *	8 (11)	8 (7)
Median CrCL (mL/min) (IQR)	52.4 (56.2)	0.0 (0.0)	52.6 (103.1)	35.1 (35.4) *	39.9 (37.2)
Median meropenem clearance (L/h) (IQR)	5.9 (4.9)	0.0 (0.0)	8.9 (15.8) *	4.9 (3.2) *	4.3 (1.7) *
Median age (years) (IQR)	72 (18)	0 (0)	46 (42) *	75 (11)	76 (7) *
Median BMI (kg/m^2^) (IQR)	28 (8)	0 (0)	30 (5)	26 (5)	26 (6) *

* *p* ≤ 0.05. BMI: body mass index; c_MER_: meropenem concentration; CrCL: creatinine clearance; SOFA: sequential organ failure assessment. Values are given as median (IQR) or as absolute numbers (N) and relative incidence (%).

## Data Availability

The datasets used and analyzed during the current study are available from the corresponding author on reasonable request.

## Supplementary Materials

The following supporting information can be downloaded at: https://www.mdpi.com/article/10.3390/antibiotics12071112/s1, Figure S1. Pharmacokinetic model for concentration simulation; Table S1. Distribution of pathogens; Table S2. Meropenem concentrations under continuous renal replacement therapy; Table S3. Empiric dosing; Table S4. Standard doses meropenem according to the German summary of product characteristics.
