# Supplementary material for: Software- and TDM-Guided Dosing of Meropenem Promises High Rates of Target Attainment in Critically Ill Patients
Source: Antibiotics (Basel). 2023 Jun 27;12(7):1112. doi: 10.3390/antibiotics12071112 (PMC10376356; doi:10.3390/antibiotics12071112)
Supplement: Supplementary file 1 [file antibiotics-12-01112-s001.zip › antibiotics-2419699-supplementary.pdf]

Supplementary Materials:

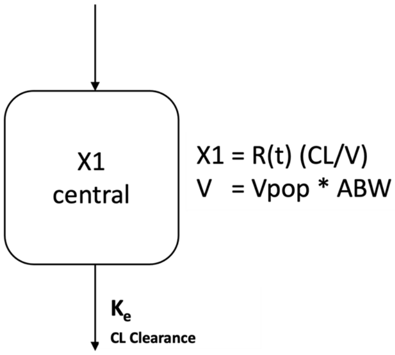

Figure S1. Pharmacokinetic model for concentration simulation. X1 describes the quantity of the drug in the central compartment, CL in L/h is the clearance of the drug from the central compartment and V in L is the volume of the central compartment, whereas V was calculated using population estimates from published literature [44] and the actual body weight (ABW).

Table S1. Distribution of pathogens.

Pathogens	*N* (%)
Escherichia coli, Proteus spp, *Klebsiella* spp	17 (19%)
ESBL Escherichia coli	5 (6%)
Enterobacter spp, *Citrobacter* spp, *Serratia* spp	9 (10%)
Pseudomonas aeruginosa, Acinetobacter spp	8 (9%)
MSSA	10 (11%)
MRSA	2 (2%)
Enterococcus faecium	3 (3%)
Other	13 (14%)

Candida spp	7 (8%)
No detection	17 (19%)

ESBL: extended-spectrum beta-lactamase; MSSA: Methicillin-susceptible *Staphylococcus aureus;* MRSA: Methicillin-resistant *Staphylococcus aureus.*

Values are given as absolute numbers (*N*) and relative incidence (%).

Table S2. Observed meropenem concentrations under continuous renal replacement therapy. Distribution of meropenem concentrations (c_{MER}) in critically ill patients with continuous renal replacement therapy (CRRT) and an individualized dosing strategy within 48 h (= c_{MER} based on software-guided empiric dosing) and after 48 h (=c_{MER} based on TDM-guided dosing). Values are given in absolute and relative incidence.

c_{MER} (mg/L)	<2	2-8	8-16	16-24	>24
Software-guided empiric dosing (*n* = 11)	0 (0.0%)	0 (0.0%)	9 (81.8%)	2 (18.2%)	0 (0.0%)
TDM-guided dosing (*n* = 10)	0 (0.0%)	1 (10.0%)	9 (90.0%)	0 (0.0%)	0 (0.0%)

c_{MER}: meropenem concentration; TDM: therapeutic drug monitoring

Values are given in absolute number (*N*) and relative incidence (%).

Table S3. Empiric dosing. Distribution of meropenem concentrations (c_{MER}) in 91 critically ill patients with a continuous infusion individualized by dosing software CADDy (= c_{MER} observed based on software-guided empiric dosing) compared to simulated continuous infusion as well as intermittent bolus administration of recommended doses according to the summary of product characteristics (=c_{MER} predicted based on standard dosing) within 48 h after onset of treatment.

c_{MER} (mg/L)	<2	2-8	8-16	16-24	>24
Predicted based on standard dosing (bolus)	16 (17.5%)	26 (28.6%)	24 (26.4%)	11 (12.1%)	14 (15.4%)
Predicted based on standard dosing (CI)	0 (0.0%)	2 (2.2%)	44 (48.4%)	25 (27.5%)	20 (22.0%)

Observed software-guided empiric dosing (CI)	0 (0.0%)	6 (6.6%)	55 (60.4%)	21 (23.1%)	9 (9.9%)

c_{MER}: meropenem concentration; CI: continuous infusion; TDM: therapeutic drug monitoring

Values are given in absolute number (*N*) and relative incidence (%).

Table S4. Standard doses meropenem according to the German summary of product characteristics.

Creatinine clearance (CrCL) (mL/min)	Daily dose meropenem (mg)*
> 50	3000
26 – 50	2000
10 – 25	1000
< 10	500

*according to the German summary of product characteristics (SmPC) of Meronem [44]
